# A case of multiple myeloma in a poultry worker

**DOI:** 10.1186/s40557-014-0035-y

**Published:** 2014-11-01

**Authors:** Pil Kyun Jung, Inah Kim, Inhyo Park, Chinyon Kim, Eun-A Kim, Jaehoon Roh

**Affiliations:** 1 Graduate School of Public Health, Yonsei University, Seoul, South Korea; 2 The Institute for Occupational Health, Yonsei University College of Medicine, Seoul, South Korea; 3 Department of Preventive Medicine and Public Health, Yonsei University College of Medicine, Seoul, South Korea; 4 Incheon Workers’ Health Center, Incheon, South Korea; 5 Occupational Safety and Health Research Institute, Korea Occupational Safety and Health Agency, Incheon, South Korea; 6 Severance Hospital, 50 Yonsei-ro, Seodaemun-gu, Seoul, South Korea

**Keywords:** Poultry, Hematologic neoplasm, Multiple myeloma, Formaldehyde, Pesticides, Solvents

## Abstract

**Background:**

Livestock breeders including poultry workers are exposed to various agricultural chemicals including pesticides and/or organic solvents. Multiple myeloma is a rare disease in Korea, and few reports have investigated the influence of occupational exposures on multiple myeloma occurrence.

**Case presentation:**

A 61-year-old male poultry farm worker presented with bone pain and generalized weakness. A bone marrow biopsy was performed, and he was diagnosed with multiple myeloma. The patient had worked in a poultry farm for 16 years and was exposed to various pesticides and organic solvents such as formaldehyde without any proper personal protective equipment. Results of the work reenactment revealed that the concentration of formaldehyde (17.53 ppm) greatly exceeded the time-weighted average (0.5 ppm) and short-term exposure limit (1.0 ppm) suggested in the Korean Industrial Safety and Health Act.

**Conclusions:**

This case report suggests that poultry workers may be exposed to high levels of various hazardous chemicals including pesticides and/or organic solvents. Numerous previous studies have suggested an association between multiple myeloma and exposure to agricultural chemicals; thus, multiple myeloma in this patient might have resulted from the prolonged, high exposure to these chemicals.

**Electronic supplementary material:**

The online version of this article (doi:10.1186/s40557-014-0035-y) contains supplementary material, which is available to authorized users.

## Background

Multiple myeloma is a type of hematological malignancy known to arise from post-germinal center plasma cells (B cells) [1]. Multiple myeloma accounts for approximately 1% of all types of malignancies and 10%–15% of hematological malignancies [2]. Epidemiologically, the median age at diagnosis is 66 years with a less than a 15% prevalence among those younger than 50 [2],[3]. Multiple myeloma is also known to occur more frequently in males and blacks [2],[4]. In Korea, multiple myeloma accounts for 0.5% of all types of malignancies and 12% of hematological malignancies [5]. The crude incidence rate (per 100,000 person-years) of multiple myeloma in Korea was found to have increased from 1999 (1.0) to 2011 (2.1) [5].

Patients who suffer from multiple myeloma present with symptoms or signs related to the infiltration of plasma cells into the relevant body organs or renal damage from excessive proteins [6]. Major symptoms or signs at presentation are anemia, bone pain, elevated creatinine, generalized weakness, hypercalcemia, and/or weight loss [6]. A diagnosis of multiple myeloma is made when serum or urinary monoclonal protein and plasma cells in the bone marrow are present with accompanying end-organ damage [7]. Several epidemiological studies have suggested that occupational or environmental risk factors of multiple myeloma include ionizing radiation [8]; petrochemicals, and organic solvents such as benzene, heavy metals, asbestos, pesticides, and herbicides; however, the results are inconclusive [9],[10]. Petrochemical, agricultural, wood, or printing workers as well as workers exposed to arsenic, lead, cutting oil, pesticides, or paints are believed to be at risk for developing multiple myeloma [11].

We present a case of multiple myeloma that occurred in a poultry farm worker who was exposed to pesticides and formaldehyde.

## Case presentation

### Patient

Sixty-one-year-old male.

### Chief complaints

Generalized weakness and bone pain.

### Past medical and family history

No specific past medical or family history.

### Occupational history

No specific occupational history.

### Present illness

The patient initially presented with bone pain accompanied by generalized weakness. The result of the urinalysis indicated signs of a urinary tract infection. Treatment with antibiotics was attempted, but without improvement. Thereafter, he was admitted for acute renal failure. Bone marrow biopsy was also performed, and the patient was diagnosed with multiple myeloma (IgG, lambda type, CRAB (−/+/+/+), ps1 iss3). Following the diagnosis, a hematologist started the patient on chemotherapy and hemodialysis.

### Occupational site and job description

The patient was the sole operator of a poultry farm, established in 1996, that had three chicken sheds in operation from 1996 to 2005 and two from 2005. The measurement of each chicken shed was 105 × 8 × 3 meters with a maximum capacity of approximately 30,000 chickens. Aside from two air intake and exhaust ventilators, six ventilator fans are present on the sidewalls of each chicken shed. The air intake and exhaust systems are used during chicken rearing periods. After the chickens mature, they are shipped out of the sheds, and then a consecutive fumigation is performed (approximately 6 times/months). In order to maximize the fumigation effect, ventilators are only turned on after the fumigation is finished, regardless of whether a worker is present in the shed at the time of the fumigation.

The patient’s tasks at the poultry farm included the management and maintenance of consumable supplies and hygiene control, although his main tasks varied depending on the chicken rearing period. During the chickens’ growth period, the patient’s job was to spray pesticides containing dichlorovinyl dimethyl phosphate or ortho-dichlorobenzene on the floors using hand sprayers to maintain hygienic conditions in the sheds. In addition, the patient performed irregular weeding around the outside of the sheds using paraquat dichloride. After the mature chickens were shipped out of the sheds, the patient was responsible for fumigating the inside of the sheds using a diesel-fuel operated, manual, combustion fog machine. Thus, the patient was exposed to various chemicals during the fumigation, and other than cotton masks and cotton gloves, he wore no other personal protective equipment. The entire fumigation process from the inside wall to the entrance of the shed takes approximately one hour. Overall, five to six fumigations were performed in two months; thus, the average time spent on fumigations was approximately 18 hours per month between 1996 and 2005 when all three sheds were in operation and 12 hours per month from 2006 to 2011 when two sheds were in operation (Additional file 1: Table S1). Therefore, the estimated time spent fumigating was approximately 3,024 total hours over the course of the patient’s employment at the farm.

### Exposure assessment

According to the materials safety data sheet, the fumigation chemicals used contain dichlorovinyl dimethyl phosphate, ortho-dichlorbenzene, cresol, methyl alcohol, benzalkonium chloride, high boiling tar acids, paraquat dichloride, sulphonic acid, chlorinated xylenols, and formaldehyde (Table 1). Previous measurements of chemicals used in the farm do not exist because the patient, who was the sole operator of the poultry farm, did not record this information.

**Table 1:** **Materials safety data sheet listing all products used in the poultry farm**

Product name	Constituent	CAS No.^*^	Percentage
Formaldehyde 35%	Formaldehyde 35%	50-00-0	100
DDVP	Dichloro vinyl dimethyl phosphate	62-73-7	90
Olsozol	Ortho-dichlorbenzene	95-50-1	N/S^†^
	M-cresol	108-39-4	N/S
	Methyl alcohol	67-56-1	N/S
Gramoxone inteon	Paraquat dichloride	1910-42-5	24
Baroclean	Benzaikonium chloride	264-151-6	50
Longlife	High boiling tar acids	84989-05-9	15 ~ 30
	Chlorinated xylenols	Mixture	N/S
	Sulphonic acid	27176-870	N/S

^*^Chemical abstracts service number.

^†^Not specified.

**Figure 1:**
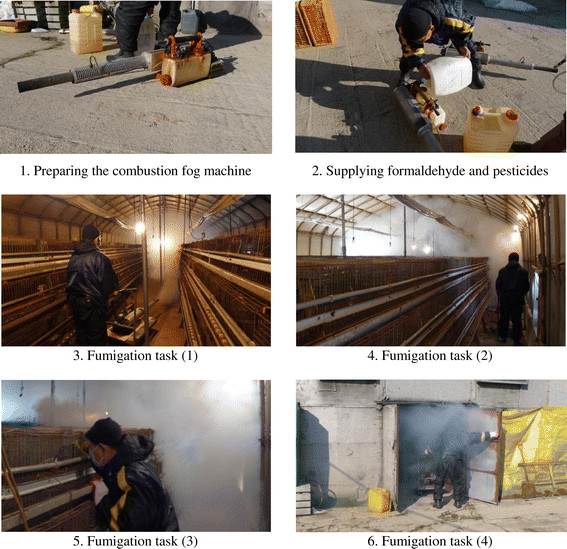
**Reenactment of the fumigation procedure for exposure measurement.**

Reenactment of the fumigation tasks were performed in January 2013 using personal samplers to estimate the levels of exposure to formaldehyde and organic solvents including benzene, which is an impurity sometimes present in pesticides (Figure 1). Samples were collected over the course of one hour. Eight-hour time weighted average exposure concentrations and the short-term exposure limit were calculated. The concentration of formaldehyde and benzene were estimated using NIOSH 2016 [12] and the 1501 methods [13]. Samples were analyzed using high-performance liquid chromatography for formaldehyde and gas chromatography with a flame ionization detector for benzene.

The results of our analysis showed only small traces of benzene in the collected samples; however, our results may not be representative of the actual concentration of benzene in the pesticides used. The concentration of formaldehyde was estimated as 17.53 ppm, which greatly exceeds the time-weighted average and short-term exposure limit of 0.5 ppm and 1.0 ppm, respectively, that are suggested in the Korean Industrial Safety and Health Act (Table 2) [14].

**Table 2:** **Results of the exposure reenactment at the farm**

Organic solvent	Method^*^	Time^†^	TWA^‡^	STEL^§^	Concentration (ppm)
Formaldehyde	Personal	13:30–14:30	0.5	1	17.53
Benzene	Personal	13:30–14:30	1	5	Trace

^*^Sampling method.

^†^Sampling duration.

^‡^Time-weighted average suggested in the Korean Industrial Safety and Health Act.

^§^Short-term exposure limit suggested in the Korean Industrial Safety and Health Act.

## Conclusions

Agricultural chemicals used in the fumigation tasks, including pesticides and formaldehyde, are suspected to be the main occupational risk factors in the current case. In 2011, 1,050 and 218,017 new cases of multiple myeloma and all types of malignancies were diagnosed in Korea, respectively [5]. Because of the rare nature of this disease, studies on the risk factors of multiple myeloma have not been able to provide conclusive evidence of potential occupational risk factors.

Considering the widespread use of agricultural chemicals such as pesticides and/or fungicides, agricultural workers, especially livestock breeders, are known to be at a high risk factor of developing hematopoietic cancer [15],[16]. Poultry workers and agricultural farmers both use a substantial amount of agricultural chemicals and organic solvents for hygiene control [16],[17].

The prevalence of multiple myeloma was higher among pesticide users than it was among other occupation groups according to the Agricultural Health Study (6.8% v. 3.7%, OR = 1.9, 95% CI: 1.3–2.7) [18]. In a case–control study conducted in Canada, the risk of multiple myeloma increased when patients were exposed to carbamates or captan class fungicides (OR = 1.90, 95% CI: 1.11–3.27, 25 cases; OR = 2.35, 95% CI: 1.03-5.35, 14 cases) [19]. In another case–control study from 2000 to 2004 in France that targeted 491 multiple myeloma patients from 6 hospitals, the risk of disease was highest among direct users of pesticides (OR = 3.5, 95% CI: 1.6–7.7, 15 cases) [20]. Several other studies have also suggested that the use of agricultural chemicals including various kinds of pesticides is related to an increased risk of multiple myeloma [21]–[23]. Working in the livestock breeding industry is also believed to be related to an increased risk of multiple myeloma especially if the livestock are sheep, cows, or pigs; however, we found no specific reports on poultry workers having an increased risk of multiple myeloma [15],[21],[24].

Among the different classes of agricultural chemicals, carbamates and organochlorides are known to be related to an increased risk of multiple myeloma [18],[25]. Previous studies have not found a relationship between the occurrence of multiple myeloma and working with chickens [21],[24]. Nevertheless, work environments can widely differ; therefore, the results of previous studies should be applied carefully [26].

Organic solvents such as benzene and formaldehyde, which our patient was exposed to, are both classified as group 1 carcinogens for hematopoietic cancer by the International Agency for Research on Cancer, and formaldehyde is a known risk factor for acute myeloblastic leukemia [27],[28]. Formaldehyde has highly reactive properties and direct damage such as aberrations on peripheral blood cells or cytogenetic damage in bone marrow cells in animals have been observed [29]. After being absorbed into the body, most of the substance is converted into an oligomer in the form of a diol such as methanediol [30]. Since the molecular weights of oligomers are small enough to penetrate biological barriers [31], formaldehyde may also promote leukemogenesis by direct DNA damage and aneuploidy in hematopoietic stem or early progenitor cells [31]–[33].

Numerous previous studies have investigated the association between exposures to organic solvents and the occurrence of multiple myeloma. In a cohort study that included formaldehyde manufacturing factory workers, increased mortality was directly proportional to the peak exposure level, yet their results were not statistically significant (peak exposure <2.0 ppm, relative risk = 1.0, 11 cases; peak exposure 2.0– < 4.0 ppm relative risk = 1.65, 13 cases, 95% CI: 0.76–3.61, p trend >0.5; peak exposure ≥4.0 ppm, relative risk = 2.04, 21 cases, 95% CI: 1.01–4.12, p trend >0.5) [34]. Studies targeting embalmers and the occurrence of other hematopoietic malignancies including myeloid leukemia also suggested heavily exposed groups were at a high risk (OR = 3.9, 95% CI: 1.2–12.5 among embalmers who worked >34 years) [27]. Few studies have investigated the influence of benzene exposure. However, one cohort study found an increased risk for hematopoietic cancers including multiple myeloma among workers exposed to unrefined petroleum chemicals containing benzene as an impurity [35],[36].

The patient in the present study had worked at the same poultry farm for the last 16 years and was in charge of managing consumable supplies and hygiene control. To maintain the hygiene of the chicken sheds, the patient performed fumigations with pesticides and formaldehyde, and ventilator fans were turned off during the fumigation process to enhance the efficiency of the fumigation. This lack of ventilation has likely led to excessive exposures to hazardous chemicals. In the reenactment of the patient’s fumigation process, levels of exposure to formaldehyde and benzene greatly exceeded the time-weighted average and short-term exposure limit suggested in the Korean Industrial Safety and Health Act [14].

As the results of the exposure reenactment indicates, the patient was repeatedly exposed to an extremely high level of formaldehyde and other agricultural chemicals including pesticides. Until now, a clear association between pesticides or formaldehyde exposure and an increased risk of multiple myeloma remain inconclusive; however, our patient’s exposure to formaldehyde was exceptionally high, thus potential risks cannot be neglected. In addition, considering the frequency and prolonged duration of exposure to these hazardous chemicals, the patient’s circumstance could be considered extraordinary. The patient presented with no other known risk factors for multiple myeloma; however, due to a lack of education on occupational safety measures, the patient wore no personal protective equipment while working.

Although this study lacks enough epidemiological evidence to establish an association with multiple myeloma, the rare nature of the disease and extremely high level of exposure to hazardous chemicals over a prolonged time leads us to believe that the patient’s multiple myeloma likely originated after occupational exposure. Most poultry workers in Korea are considered petty; therefore, exposure to numerous health hazards is common, and they tend to receive no proper education on protective measures. In the future, improvements to work environments and educational programs on occupation hygiene are required. Moreover, the health effects of agricultural chemicals should be further evaluated. Last, large-scale prospective studies tracking the concentrations of compound chemicals used in agricultural work are required, and techniques to reduce their health effects should be developed.

## Consent

Written informed consent was obtained from the patient for the publication of this report and all accompanying images.

## Additional file

## Electronic supplementary material

Additional file 1: Table S1.: Typical work schedule of the poultry farm. (DOC 36 KB)

Below are the links to the authors’ original submitted files for images.

Authors’ original file for figure 1
