# Supplementary material for: A case of multiple myeloma in a poultry worker
Source: Ann Occup Environ Med. 2014 Nov 1;26:35. doi: 10.1186/s40557-014-0035-y (PMC4279810; doi:10.1186/s40557-014-0035-y)
Supplement: Supplementary file 1 — Additional file 1: Table S1.: Typical work schedule of the poultry farm. (DOC 36 KB) [file 40557_2014_9035_MOESM1_ESM.doc]

Additional file 1: Table S1 **Typical work schedule of the poultry farm**

| **Day 1** Arrival of the chickens | **Day 2** | **Day 3** | **Day 4** Floor Disinfection | **Day 5** | **Day 6** | **Day 7** |
| --- | --- | --- | --- | --- | --- | --- |
| **Day 8** Floor Disinfection | **Day 9** | **Day 10** | **Day 11** | **Day 12** Floor Disinfection | **Day 13** | **Day 14** |
| **Day 15** | **Day 16** Floor Disinfection | **Day 17** | **Day 18** | **Day 19** | **Day 20** Floor Disinfection | **Day 21** |
| **Day 22** | **Day 23** | **Day 24** Floor Disinfection | **Day 25** | **Day 26** | **Day 27** | **Day 28** Floor Disinfection |
| **Day 29** | **Day 30** | **Day 31** | **Day 32** Floor Disinfection | **Day 33** | **Day 34** Shipment of the mature chickens | **Day 35** Shed fumigation |
| **Day 36** Shed fumigation | **Day 37** | **Day 38** | **Day 39** Shed fumigation | **Day 40** Shed fumigation | **Day 41** | **Day 42** |
| **Day 43** Shed fumigation | **Day 44** Shed fumigation | **Day 45** | **Day 46** | **Day 47** Shed fumigation | **Day 48** Shed fumigation | **Day 49** |
| **Day 50** | **Day 51** Shed fumigation | **Day 52** Shed fumigation | **Day 53** | **Day 54** | **Day 55** Shed fumigation | **Day 56** Shed fumigation |
| **Day 57** | **Day 58** | **Day 59** | **Day 60** Arrival of new chickens | **Day 61** | **Day 62** | **Day 63** Floor disinfection |
