# Supplementary figures and images for: A case of multiple myeloma in a poultry worker
Source: Ann Occup Environ Med. 2014 Nov 1;26:35. doi: 10.1186/s40557-014-0035-y (PMC4279810; doi:10.1186/s40557-014-0035-y)

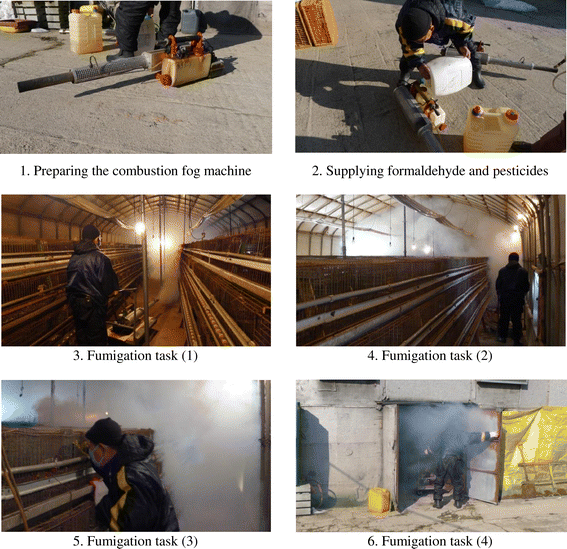

Supplement: Supplementary file 2 — Authors’ original file for figure 1 [file 40557_2014_9035_MOESM2_ESM.gif]
